# Delayed Development of Trigeminal Neuralgia after Radiosurgical Treatment of a Tentorial Meningioma

**DOI:** 10.7759/cureus.1628

**Published:** 2017-08-30

**Authors:** Aldo Berti, Michelle Granville, Xiaodong Wu, David Huang, James G Schwade, Robert E Jacobson

**Affiliations:** 1 Miami Neurosurgical Center, University of Miami Hospital; 2 Innovative Cancer Institute; 3 Cyberknife Center of Miami, University of Miami Miller School of Medicine

**Keywords:** trigeminal neuralgia, tentorial tumor, stereotactic radiosurgery, cyberknife radiosurgery, postradiosurgery complications, childhood whole brain radiation, radiation dosage, tentorial meningioma, radiation induced meningioma

## Abstract

Trigeminal neuralgia is a known symptom of the tumors and aberrant vessels near the trigeminal nerve and the tentorial notch. There are very few reports of delayed development of trigeminal neuralgia after radiosurgical treatment of a tumor in these areas. This is a case report of a patient treated with radiosurgery for radiation induced meningiomas, 30 years after childhood whole brain radiation. The largest tumor was adjacent to the pons and left trigeminal nerve but did not cause any direct neurologic symptoms or facial pain. Nine months after radiosurgical treatment of the tumors, the patient developed left sided typical trigeminal facial pain and magnetic resonance imaging (MRI) demonstrated the marked reduction in the tumor size. The patient was subsequently treated with radiosurgery to the Gasserian ganglion with a resolution of facial pain. This article reviews the unique characteristics and unusual response to the radiation induced meningiomas to radiosurgery. This is a case of rapid shrinkage of the tumor seen on follow-up MRI scans, concurrent with the development of facial pain, suggests that the rapid shrinkage led to traction on adhesions and related microvasculature changes adjacent to the tumor and trigeminal nerve roots causing the subsequent trigeminal neuralgia.

## Introduction

Trigeminal neuralgia can be caused by structural compression from aberrant vessels such as the superior cerebellar artery and tumors that are adjacent to the trigeminal ganglion, Meckel’s cave and the brainstem. These patients commonly present with trigeminal pain or numbness in the face [1-3]. This is a case report of a patient who developed two meningiomas that were initially detected 25 years after childhood whole brain radiation when a magnetic resonance imaging (MRI) scan was performed for intermittent headaches. The larger tumor was adjacent to the left tentorial notch and Meckel's cave, while the second smaller tumor was involving the posterior right cavernous sinus. Serial MRI scans over a period of four years demonstrated progressive enlargement of both tumors. The patient was then treated with stereotactic radiosurgery for both the tumors. Nine months after radiosurgery, the patient developed the first symptoms of left trigeminal neuralgia, while at the same time, the MRI scans showed the left tentorial notch tumor to be significantly reduced in size. In this case, the delayed onset of trigeminal neuralgia developed from a mixture of rapid reduction in tumor size after radiosurgery, plus adhesive traction on the trigeminal nerve and root entry zone. The abnormal cytopathology identified during radiation induced tumors may be a factor in the rapid tumor response to stereotactic radiosurgery [4-5]. Therefore, the delayed development of multiple meningiomas after childhood whole brain radiation and the need for continual long term follow-up in such patients, as well as the unique pathologic findings in radiation induced tumors and their sensitivity to radiation is reviewed in this case. Informed consent was obtained from the patient for this study.

## Case presentation

**Figure 1:**
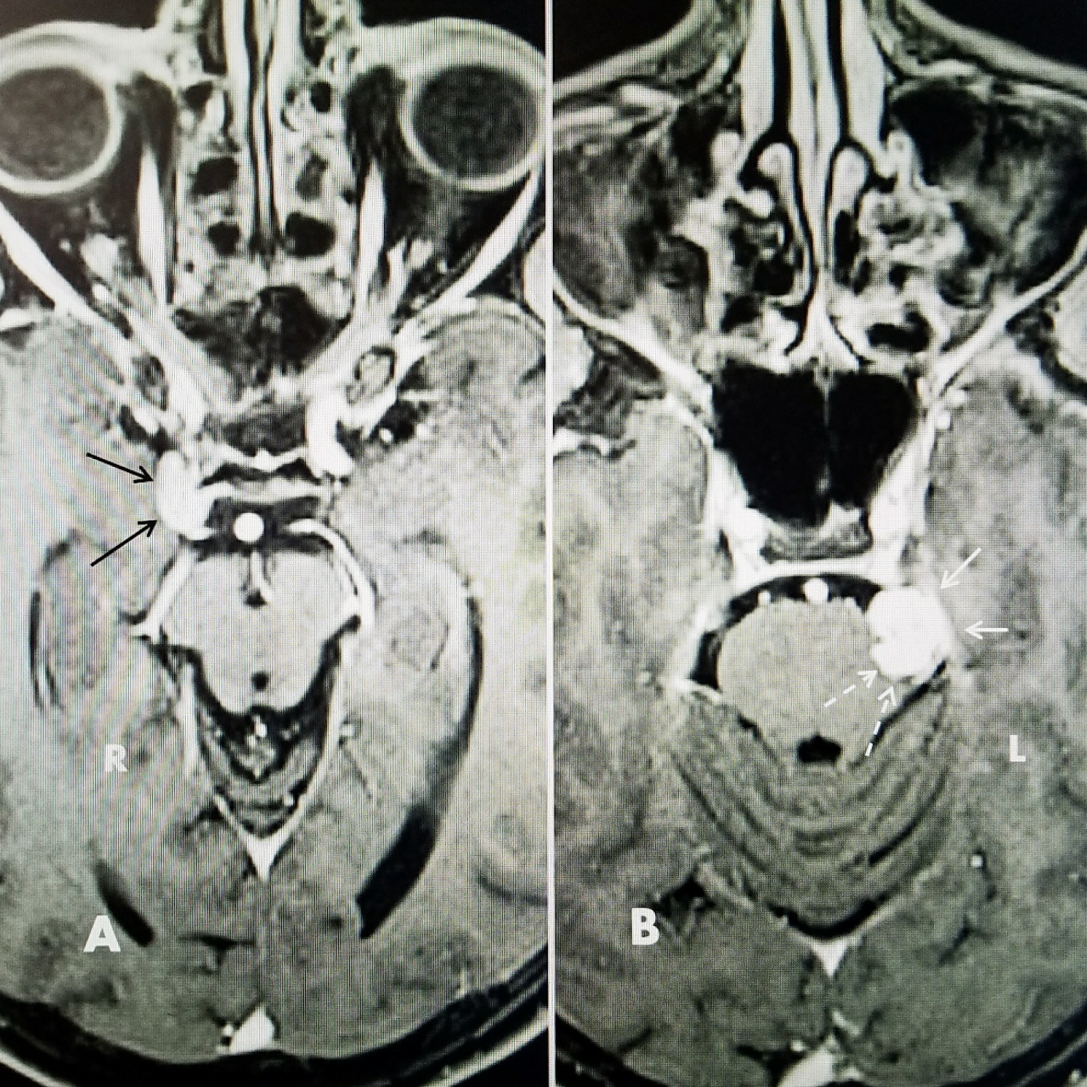
Magnetic resonance imaging T1 MRI with gadolinium showing right cavernous sinus meningioma and left tentorial notch tumor. A: Axial T1 MRI with contrast: showing right (R) cavernous meningioma (black arrows). B: Axial T1 MRI with contrast showing left (L) bilobed irregular tentorial mass indenting pons in left prepontine and trigeminal cistern. White arrows show smooth attachment to tentorium. The dotted white arrows medially show the relationship of the tumor to trigeminal root entry zone when the patient had no trigeminal pain.

This is a case of a 54-year-old male who was treated at 13 years of age with a combination of chemotherapy, bone marrow transplant, and whole brain and spinal axis radiation (cranial 24 Gray) spinal (12 Gray-Gy) for acute lymphocytic leukemia (ALL). At the of age 46 years, while the patient was being evaluated with MRI scans for intermittent headaches, it was discovered that the patient had developed two intracranial tumors radiologically consistent with meningiomas. The tumors were a right cavernous sinus tumor of 13 x 10 millimeters and a larger multilobulated left tentorial tumor of 13 x 11 x 12 mm with direct compression on the left pons. The repeat MRI scans over a period of five years demonstrated the continued growth of the tumors, especially in the left tentorial notch. At the age 51 years, although the patient had no neurologic symptoms, because of continued tumor growth, he was referred for radiosurgical treatment. At this time, the right cavernous sinus tumor was 19 x 14 mm with a tumor volume of 792 mm^3^ and contiguous to the right anterior brainstem, while the left tentorial tumor was more lobulated and 24 x 14 mm with a tumor volume of 2002 mm^3^. There was marked direct indentation and pressure on the left lateral pons, including the root entry zone of the left trigeminal nerve (Figure 1).

**Figure 2:**
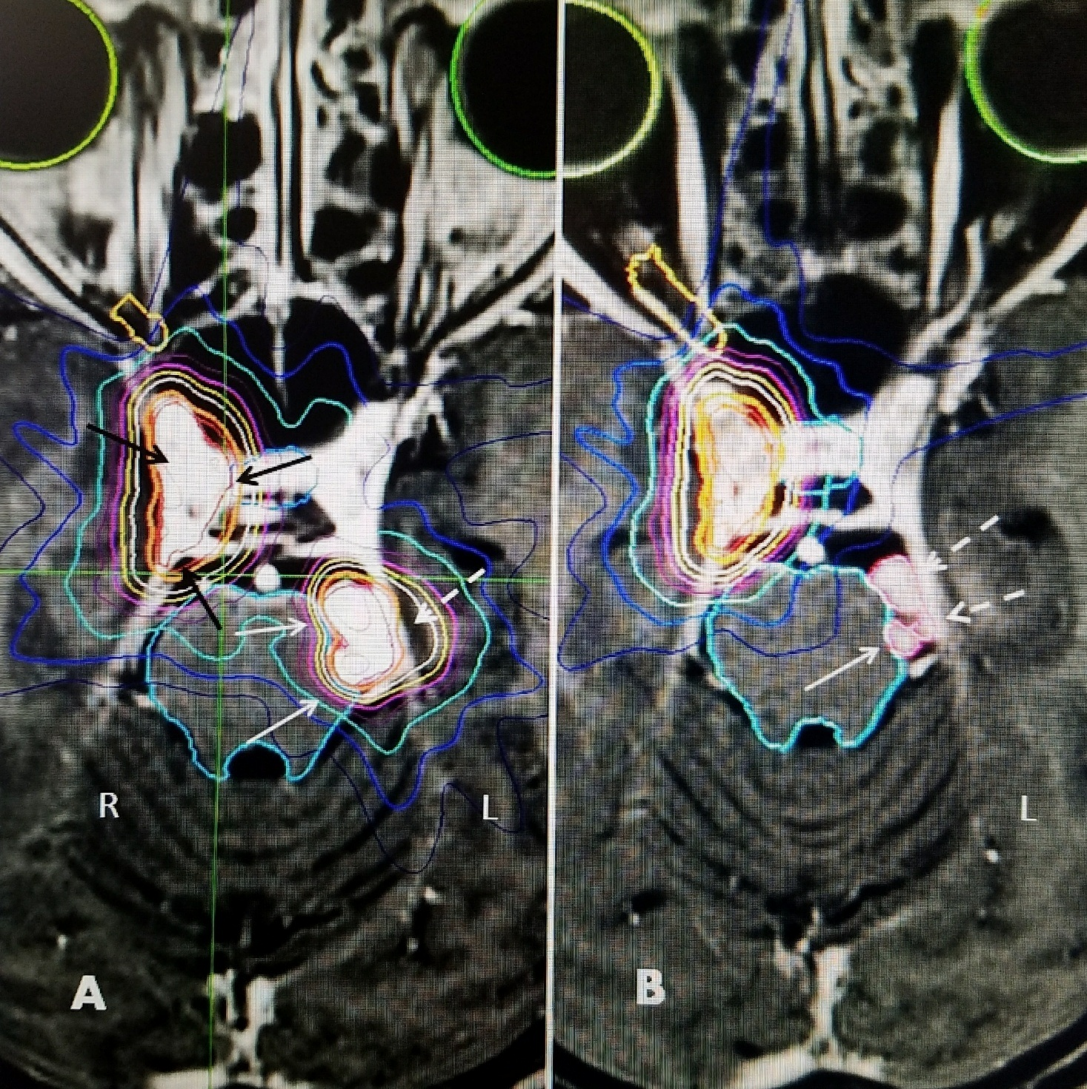
Original radiosurgical plan showing right cavernous sinus and left tentorial meningiomas. A: Axial radiosurgical plan at the level of right (R) cavernous meningioma (black arrows) and left (L) bilobed tentorial meningioma indenting pons (white arrows) and smooth broad attachment to tentorium (dotted white arrows). B: Slightly more inferior part of plan showing small lobular parts of the tumor against pons on left. The full coverage of the right cavernous sinus tumor by stereotactic is seen.

Both the tumors were treated in three sessions of radiosurgery using the Cyberknife^R ^(Accuray^R^, Sunnyvale, California, USA) with the right cavernous sinus/posterior-clinoid tumor receiving 12 Gy to the 77% isodose line and the left tentorial tumor receiving 18 Gy to the 85% isodose line in three fractions of 600 cGy each (Figure 2).

**Figure 3:**
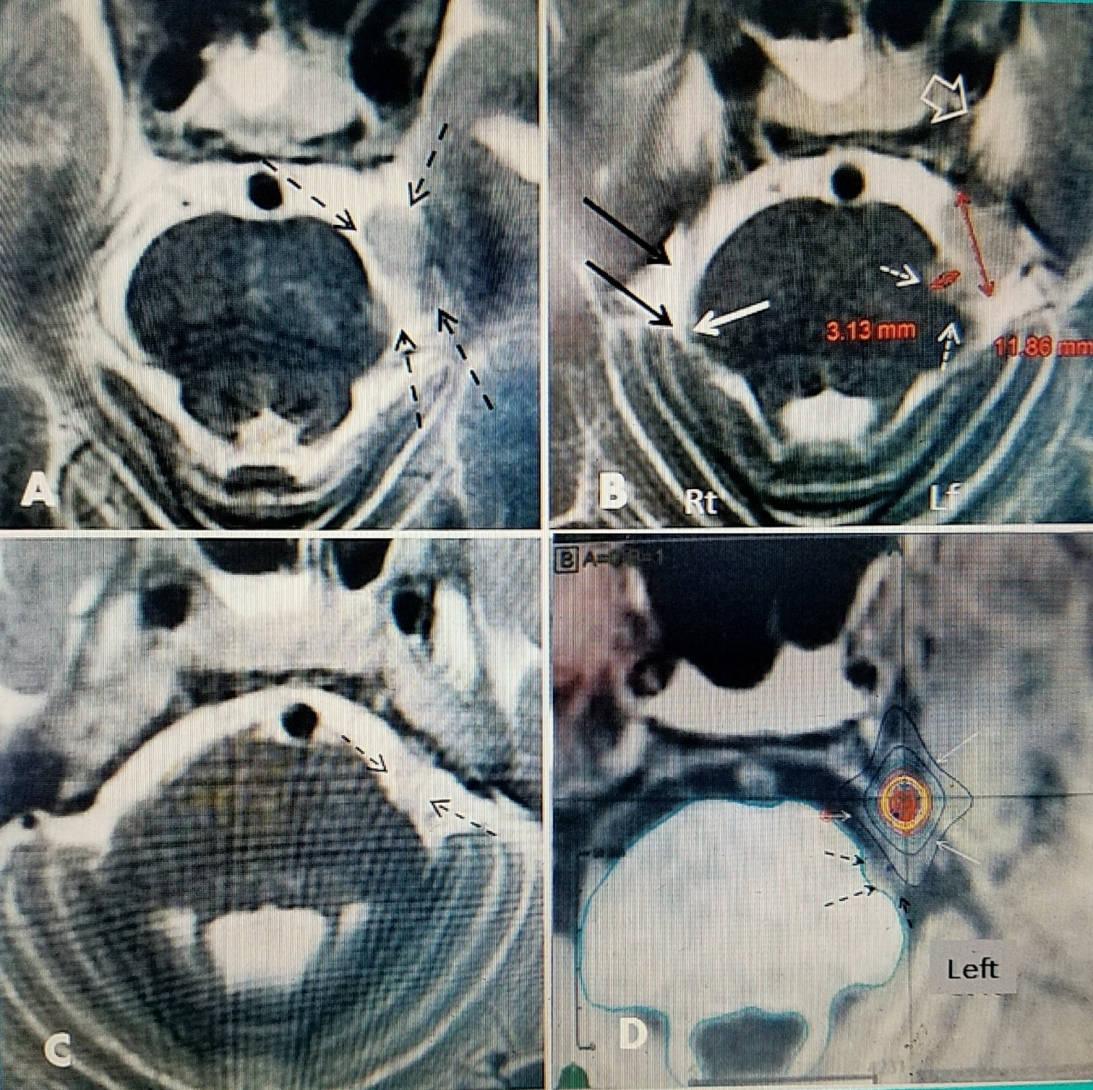
The 24-month follow-up scans post original treatment and plan for radiosurgery to the trigeminal nerve. Axial 3.0 Tesla T2 POST scans​ A: There is a minimal contact of the tumor with fifth cranial nerve root entry zone (dotted black arrows). The tumor is in the lateral pontine cistern. B: Residual tumor is in the lateral pontine cistern (the left trigeminal ganglion cistern and Meckel's cave is denoted by large white arrow), the tumor is 11.88 mm in sagittal dimension but there is a tumor nodule of 3.13 adjacent and indenting the lateral pons (horizontal red arrow) very near the fifth nerve root entry zone (dotted white arrows). The tumor is in the lateral pontine cistern and not in the more anterior prepontine extension or into the trigeminal cistern. The normal right fifth nerve ( black arrows) and root entry zone (solid white arrow) is seen. C: Shadow of left fifth nerve deflected by the tumor as it enters pons, at the level of the forth ventricle, from the trigeminal cave (dotted black arrows). D: Radiosurgical plan for 70 gray (Gy) treatment to Gasserian ganglion at left Meckel's cave. The border of residual tumor adjacent to root entry zone for trigeminal nerve is seen (dotted black arrows). The plan was shaped to specifically avoid the brainstem, oriented along the long axis of the trigeminal ganglion (fine white arrows).

Nine months after radiosurgery to the tumors, the patient started developing lancinating left facial pain without numbness. He initially responded to medical management with carbamazepine 100 mg and gabapentin 300 mg daily. However, gradually over the next 15 months, his pain recurred and it was unresponsive to the medical treatment, despite increasing doses of carbamazepine to 600 mg daily and gabapentin 1800 mg daily. With high doses of carbamazepine and gabapentin needed to control his facial pain, he developed side effects which left him unsteady and somnolent. The repeat MRI scans showed the left tentorial tumor had reduced to 11.86 mm. The MRI scan, 24 months after original tumor treatment revealed 3.13 mm residual tumor adherent to the left fifth cranial nerve root entry zone with no tumor in the trigeminal cistern. He did not want to consider balloon compression and underwent another radiosurgery treatment of 70 Gy to the left Gasserian ganglion. He responded with loss of pain and was on baseline medication of gabapentin 300 mg two times daily and carbamazepine 100 mg daily (Figure 3). 

## Discussion

Trigeminal neuralgia secondary to the tumors

The majority of cases of trigeminal neuralgia have no known etiology; however, in a specific group of patients, it can be caused by compression of aberrant vessels such as the superior cerebellar artery, the tumors adjacent to the trigeminal ganglion, Meckel’s cave and the brainstem. During an evaluation, in a large series of patients presenting with facial pain, the reported incidence due to adjacent tumors varied from 2-11% [1]. There are numerous single case reports of the tumors in the vicinity of the tentorial notch near the trigeminal nerve presenting with either atypical facial pain or numbness in the facial area rather than episodic trigeminal neuralgia. Typically these are benign tumors such as epidermoid, meningiomas, or vestibular schwannomas and rarely temporal lobe gliomas pressing into the tentorial notch [2-5]. Most of these tumors are on the same side where the proximity of the tumor to the trigeminal ganglion and nerve causes direct compression. Epidermoid tumors often develop actual encasement of the nerve by the tumor [2,4] .There are also several case reports of contra-lateral trigeminal neuralgia explained by a shift of the brain stem causing traction on the opposite trigeminal ganglion after the tumor resection. This case is similar in where tumor shrinkage caused traction on the trigeminal ganglion. From direct surgical observation, it is postulated trigeminal neuralgia may develop secondary to post-surgical adhesions [2-3]. Relative to this specific case, the development of gliomas and meningiomas have been recognized as a major but very delayed complication of childhood whole brain radiation [4-5]. Gliomatous tumors are more commonly found in the first five years after whole brain radiation, while meningiomas are typically found after a very latent interval of 20 or more years. In a large series with long term follow-up of post childhood radiation, when intracerebral tumors occurred, they were often in multiple locations. The incidence of the tumors progressively increases during the length, the patient survives. As a result, in patients after childhood whole brain radiation, the tumor growth is a continual potential threat, often growing asymptomatically for many years, as in this case, but once the tumors are identified, they continue to increase in size unless treated [4]. Although these tumors are documented to be slow growing, the tumors are found to be histologically atypical, more aggressive, have a higher recurrence rate and a higher histopathological grade with complex cytogenetic aberrations compared to non-radiation induced meningiomas [5].

Stereotactic radiation for the tumor reduction

The rapid tumor response to radiosurgery with marked shrinkage of the tumors within nine months is an atypical course for the radiosurgical response for meningiomas. Such rapid tumor response would be seen with aggressive atypical tumor cells with a higher histopathologic grade [5]. This can be an explanation for the marked tumor reduction in nine months after stereotactic radiation. Rapid diminution in the tumor size causing traction on chronic adhesions to the trigeminal nerve and rootlets entering the brainstem or displacement of the trigeminal nerve complex is the most likely explanation for the development of trigeminal neuralgia. Normally, in cases of radiosurgical treatment of trigeminal neuralgia directly related to an underlying tumor, the radiosurgical dose is directed at the tumor rather than the trigeminal ganglion or nerve [2-4]. Because trigeminal nerve is directly under the radiated tumor, it is unclear if that original 18 Gy dose to the tumor had any secondary effect on the underlying trigeminal nerve and ganglion. 

Considerations for repeat and subsequent radiation dosage

The use of additional radiation in the patients who had previous whole brain radiation or other stereotactic treatments is possible as long as the total lifetime cumulative effective dosage is under 116-137 Gy. The exact whole brain dose to the patient received 30 years earlier at age 13 years was unknown, but at the time of treatment, the typical dose to the brain and axis was fractioned at 40 Gy in 2 Gy increments. Studies show there is less risk of radio-necrosis and the dose is more tolerated with smaller volumes treated and with fractioned or stereotactic doses such as radiosurgery limited to several millimeters of the trigeminal ganglion, as was done in this patient [6]. There are reports of multiple radiosurgical treatments for trigeminal neuralgia without adverse effects [7]. For the treatment of the meningiomas, the patient received highly focused stereotactic doses based on volumes of 792 mm^3^ and 2002 mm^3^ of 12 and 18 Gy to the tumors. Fifteen months later, the patient received 70 Gy to the left trigeminal ganglion to a volume fewer than 0.17 mm^3^. In the radiosurgical plans, there was none or minimal spread to the adjacent neural tissue with attention being carefully paid to avoid the contiguous brain stem [8]. There was no evidence of numbness in the face indicative of permanent damage to the trigeminal ganglion secondary to the tumor or radiation effect [8]. Studies of the trigeminal ganglion and root entry zones using MRI and magnetic resonance angiogram, plus diffusion tensor imaging have demonstrated microstructural changes and axonal loss within the trigeminal nerve in spontaneous cases of trigeminal neuralgia not associated with the tumors, as well as showed more prominent venous and arterial changes post treatment [9-10]. In this case, it is probably a combination of initial rapid tumor shrinkage, adhesions to the trigeminal ganglion and root entry zone, and microvascular changes around the ganglion and the tumor bed that led to the trigeminal pain.

## Conclusions

In conclusion, the development of delayed onset of trigeminal neuralgia nine months after stereotactic radiosurgery is uncommon. It is clear that radiation induced the tumors not only have atypical histologic characteristics, but possibly has a different response to stereotactic radiation resulting in a rapid tumor reduction rather than the slow response seen with typical meningiomas. The development of trigeminal pain from rapid tumor shrinkage is an unusual cause of trigeminal neuralgia. The related factors, in this case, are chronic adhesions causing traction in the trigeminal ganglion, trigeminal rootlets and root entry zone, and associated neurovascular effects on the trigeminal ganglion nerve. Furthermore, this case also highlights the long-term risk of cerebral tumor growth after childhood whole brain radiation and the need to continuously monitor these patients for later tumor growth.
